# The Malaria Vaccine Implementation Programme study area in Ghana: results of a household survey prior to the introduction of the RTS,S/AS01 vaccine

**DOI:** 10.1186/s12936-025-05778-9

**Published:** 2026-01-12

**Authors:** Paul Welaga, Thomas Gyan, Kwadwo Koram, Abraham Hodgson, Eliezer Odei-Lartey, Francis Agbokey, Stephaney Gyaase, Elizabeth Awini, Dennis Adu-Gyasi, Gregory K. Amenuvegbe, Dora Dadzie, Abdul-Razak Adam, John Hammond, Augustine Sarfo, Abdul-Razak Nuhu, Peter Takyi Peprah, Lucy Twumwaa, Emmanuel Boateng, Paul Snell, Ari Fogelson, Rafiq N. A. Okine, Mary J. Hamel, Paul Milligan, Kerryn A. Moore, Fred N. Binka, Edwin A. Afari, Kwaku Poku Asante

**Affiliations:** 1 University of Technology and Applied Sciences, P. O. Box 24, Navrongo, Ghana; 2 Research and Development Division, Ghana Health Service, Navrongo Health Research Centre, Navrongo, Ghana; 3 Research and Development Division, Ghana Health Service, Kintampo Health Research Centre, Kintampo North Municipality, Bono East Region, Ghana; 4 Noguchi Memorial Institute for Medical Research, University of Ghana, Accra, Ghana; 5 Research and Development Division, Ghana Health Service, Accra, Ghana; 6 Research and Development Division, Ghana Health Service, Dodowa Health Research Centre, Greater Accra Region, Dodowa, Ghana; 7 FN Binka School of Public Health, University of Health and Allied Sciences, Ho, Ghana; 8 School of Public Health, University of Ghana, Accra, Ghana; 9 Ghana Health Service, Bono Regional Health Directorate, Sunyani, Ghana; 10 Ghana Health Service, Central Regional Health Directorate, Cape Coast, Ghana; 11 Ghana Statistical Service, Accra, Ghana; 12 London School of Hygiene and Tropical Medicine, London, UK; 13 Faculty of Epidemiology and Population Health, London School of Hygiene and Tropical Medicine, London, UK; 14 Global Malaria Programme, World Health Organization, Geneva, Switzerland; 15 Department of Immunizations, Vaccines, and Biologicals, World Health Organization, Geneva, Switzerland

**Keywords:** Malaria vaccine pilot evaluation, Immunization, Health-seeking behaviours, Ghana

## Abstract

**Background:**

In 2019, the RTS,S/AS01_E_ malaria vaccine (RTS,S) was introduced into Ghana’s routine health system as part of the Malaria Vaccine Implementation Programme (MVIP). Household surveys were conducted prior to vaccine introduction and approximately 18 and 30 months post-introduction. We present a description of the area in Ghana based on the baseline household survey including malaria prevalence, malnutrition, wealth, insecticide-treated net (ITN) coverage, other health interventions (deworming, Vitamin A supplementation (VAS)), coverage of Expanded Programme on Immunization (EPI) vaccines, and health-seeking behaviour for febrile children.

**Methods:**

The baseline household survey was conducted between 25 February and 18 March 2019 in a representative sample of 6778 households across 66 districts (33 in each of the implementing and comparator areas) in Ghana. Caregivers of children aged 5–48 months were interviewed. For each child, vaccination details were transcribed from the maternal and child health record book, and we measured the mid-upper arm circumference and obtained a malaria Rapid Diagnostic Test (RDT). Survey-weighted coverage estimates were obtained using standard survey methods. Survey Poisson regression was used to estimate prevalence ratios.

**Results:**

Overall, 7768 children were included in the study, and 21% (95% CI 18–23) tested positive for malaria parasitemia by RDT. About 87%, 95%CI (85–89) of all households owned at least one ITN, and 62%, 95%CI (59–64) of children aged 5–48 months slept under an insecticide-treated net (ITN) the night before the survey. Additionally, 22%, 95%CI (21–24) of children reported having fever in the two weeks preceding the survey; among those with reported fever, 72%, 95%CI (69–74) sought advice or treatment, 40%, 95%CI (37–44) were tested for malaria, and 42%, 95%CI (39–46) of those with fever took an antimalarial drug. Additionally, 17%, 95%CI (16–19) had a mid-upper arm circumference (MUAC) ≤ 13.5 cm, and 1%, 95%CI (0–1) had a (MUAC) ≤ 11.5 cm. The uptake of vitamin A VAS in the 6 months prior to the survey was 36%, based on routine delivery through EPI, and deworming coverage was 29%. Coverage of EPI vaccines was > 90%. Indicators in comparison and implementation areas were comparable.

**Conclusions:**

The pilot implementation and evaluation of the RTS,S malaria vaccine in Ghana was conducted in an area with substantial malaria transmission and illness, modest health-seeking behaviour and ITN use, and good EPI vaccine coverage. This study has established the baseline comparability between implementation and comparator areas, which serves as the foundation for future feasibility assessments.

**Supplementary Information:**

The online version contains supplementary material available at 10.1186/s12936-025-05778-9.

## Background

Of the 263 million malaria cases reported worldwide in 2023, an estimated 94% occurred in Africa [1]. About 30% of all malaria cases occur in children under five years of age in Ghana [2], and in 2022, Ghana accounted for 2.1% of global malaria cases and 4% in West Africa. In Ghana, the disease burden has remained stable for two years, at 165 cases per 1000 of the population at risk between 2020 and 2021, with a stable incidence of malaria deaths (0.39 and 0.38 per 1000 of the population in 2020 and 2021, respectively), despite sustained efforts to control malaria [3]. Malaria accounted for 41% of outpatient cases and 18% of hospital admissions among children and pregnant women in 2020 [4]. To reduce the burden of malaria, several interventions have been scaled up. These include expanding access to insecticide-treated nets (ITNs) through mass campaigns every three years. The last ITN campaign was conducted in 2018, a year prior to the implementation of the survey. ITN coverage (defined as the proportion of households with at least on ITN) has increased from 30% in 2008 to 67% in 2019, and ITN usage (defined as the proportion of children who slept under an ITN the night before the survey day) from 21 to 43% within the same period [5]. Other interventions implemented included the provision of at least three doses of sulfadoxine-pyrimethamine (SP) for intermittent preventive treatment in pregnancy (IPTp) in malaria endemic areas, indoor residual spraying, and the implementation of seasonal malaria chemoprevention (SMC) in some parts of the country including the malaria vaccine pilot evaluation (MVPE) area.

Ghana plans to eliminate malaria through progressive control using a sub-national approach, deploying a mix of cross-cutting malaria interventions tailored to the local context across the country. These interventions include quality case management of malaria, ensuring availability and rational use of quality malaria commodities, high ITN coverage, larval source management (LSM), entomological surveillance, epidemiological surveillance, research, social behaviour change (SBC), effective programme leadership and management at all levels, and malaria vaccination [6].

Despite the widespread implementation of malaria interventions in Ghana, malaria transmission remains intense, with children and pregnant women at appreciable risk [7]. In 2015, the European Medicines Agency issued a positive scientific opinion following a review of data from a Phase 3 trial of the RTS,S/AS01E malaria vaccine (RTS,S) [8]. Following this, the World Health Organization (WHO) then considered the potential use of RTS,S and recommended a large-scale pilot implementation of the four-dose regimen in children aged at least 5 months at the first dose in settings with moderate-to-high malaria transmission in Ghana, Kenya, and Malawi [9]. The criteria for selecting these countries for the pilot implementation included having moderate to high malaria transmission, functioning malaria and immunization programmes, and good coverage of EPI vaccines and malaria interventions.

**Fig. 1:**
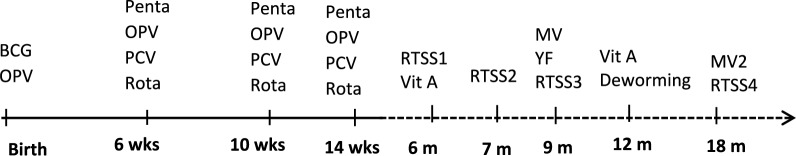
Vaccination schedule in Ghana*Note, BCG: Bacillus Calmette-Guérin; OPV: Oral Polio Vaccine; PCV: Pneumococcal Conjugate Vaccine; Penta: Pentavalent vaccine; Rota: Rotavirus vaccine; MV: Measle vaccine; YF: Yellow fever vaccine; Vit A: Vitamin A

Ghana launched its Expanded Programme on Immunization (EPI) in June 1978 with six antigens: BCG, measles, diphtheria‐pertussis‐tetanus (DPT), and oral polio for children under one year of age, together with tetanus toxoid (TT) vaccination for pregnant women [10]. Since then, new vaccines have been added, and the EPI schedule currently includes 14 antigens (Fig. 1). Ghana health authorities introduced RTS,S in selected districts in seven of the sixteen regions, with doses scheduled at 6, 7, 9, and 24 months. Doses at 9 months, therefore, correspond with an existing EPI contact point for the first dose of measles and yellow fever, while doses at 6, 7, and 24 months required new contact points. As of 2021, the schedule for RTS, S dose 4 delivery at 24 months was changed to 18 months to correspond with the second dose of the measles-rubella vaccine.

Immunization services are delivered through three strategies: static at health facilities, outreach in the communities, and campaigns. The feasibility assessment of delivering a 4-dose schedule was based on RTS,S coverage estimated in a series of household surveys. The baseline household survey was designed to generate estimates of the routine EPI vaccination coverage, the coverage and utilization of recommended malaria control measures such as the use of ITN, indoor residual spraying (IRS), and to document the health seeking behavior for febrile children before the pilot implementation of the RTS,S malaria vaccine in Ghana. The baseline household survey estimates of these and other selected health indicators are presented for children aged 5–48 months in Ghana.

## Methods

### Survey design

The baseline household survey was conducted between February 25 and March 18, 2019, in 66 districts (33 in each of the implementing and comparator areas) across six regions of Ghana. Mothers or caregivers of children aged 5–48 months were interviewed using a standardized questionnaire. Dates of all vaccinations were transcribed from the maternal and child health record book. The mid-upper arm circumference (MUAC) of the children were measured, and each child was tested for the presence of malaria parasitaemia using a rapid diagnostic test (RDT).

### The study setting

**Fig. 2:**
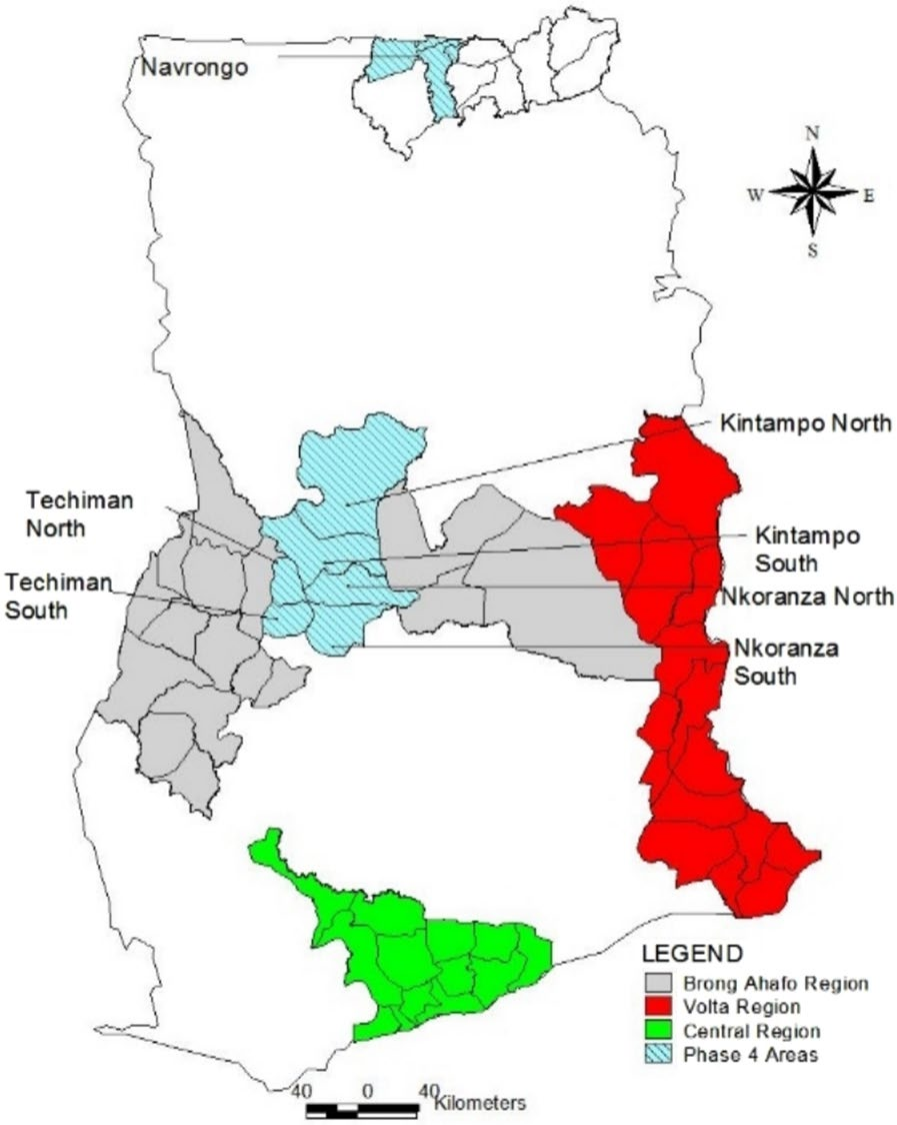
A map of Ghana showing the evaluation areas for the MVIP, 2019

The baseline household survey was conducted in formerly three, now six, of the Malaria Vaccine Implementation Pilot (MVIP) regions of Brong Ahafo (now Bono, Bono East, and Ahafo), Central, and Volta (now Volta and Oti) regions of Ghana. These regions were selected by the Ghana Health Service and the Ministry of Health for the MVIP based on the burden of malaria morbidity and existing immunization infrastructure. The estimated total population in the six regions was 8.2 million, with 49% males and 51% females [11] (Fig. 2).

### Sample size

A sample size of 100 households per cluster, or 6600 households across the 66 clusters involved in the pilot, was expected to estimate cluster-specific coverage of interventions to within 10% (i.e. 95% CI from 40 to 60%) of a true value of 50% coverage if response rate was above 95% in each cluster.

### Sampling

Four enumeration areas (EAs) with probability proportional to population, from the 2010 Population and Housing Census sampling framework used by the Ghana Statistical Service (GSS), were selected in each district. Each selected EA, was divided into segments of approximately equal population, using EA maps provided by the GSS, to obtain at least 25 households with children aged 5–48 months in each segment. One segment was then randomly selected, and all households within the selected segment were visited to identify and interview all eligible participants. All visited households were marked to prevent multiple visits to the same household by interviewers and for quality visits by study monitors.

### Community sensitization

Before the survey began, community members and opinion leaders, including assembly members, were provided with information on the survey activities. Letters were written to the regional health directorates and the district assemblies in the pilot implementation regions to notify them of the survey and solicit their support. The Ghana Health Service, through the EPI, led community sensitization efforts.

### Recruitment and training of data collectors

Research assistants with graduate-level training were recruited and trained for 5 days on the study design, sampling procedures, and how to obtain informed consent and administer the questionnaire. They were also trained in malaria testing using RDTs and to measure the MUAC of children. The tools were pretested in communities not included in the sampled EAs for the survey and revised accordingly after the pretest.

### Questionnaire

Interviewers administered a questionnaire for the survey. Questions included the background characteristics of households, respondents and children; vaccination coverage; coverage of malaria interventions (ITN/LLIN, IRS); use of ITNs; household socio-economic status; patterns of health-seeking behaviours for febrile children, including seeking malaria testing and treatment; other childhood interventions such as deworming and vitamin A supplementation, and nutritional assessment.

To ensure comparability of our results with previous surveys and MVPE data from other countries, some questions used in the Ghana Demographic and Health Surveys (GDHS), Malaria Indicator Surveys (MIS), and Multi-Indicator Cluster Surveys (MICS) were adapted.

### Data collection

Data collection was carried out by 21 field teams, each comprising one supervisor, four interviewers, and one driver. Each team was assigned a number of EAs in a region. Researchers and staff from the GSS coordinated and monitored the fieldwork activities. In each household, mothers or caregivers of eligible children aged 5–48 months were interviewed. If a caregiver or an eligible child was absent at the time of the visit, the interviewer made two revisits to the household. Supervisors observed and supported interviewers to ensure the quality of the collected data was improved and maintained. This was achieved by observing interviews, verifying the consent forms, and continuously reviewing challenges with the interviewers. Study monitors visited the field team to resolve field challenges and conduct quality control checks.

### Data management

The data were electronically captured and managed using REDCap application, hosted at the Kintampo Health Research Centre [12]. After a supervisor checked for missing entries and inconsistencies, the records of each interviewer were uploaded into a database server. The data management team generated daily reports to assess the quality of the submitted data, checking the key missing variables and duration of the interviews. Data editing and cleaning were performed to identify outliers and verify inconsistencies, ensuring the accuracy and completeness of the work in the field. Errors detected during quality control checks were communicated to the field team for resolution and correction.

### Data analysis

A harmonized analysis of baseline survey data from the three MVPE countries was carried out using STATA 18.0. A wealth index was computed using principal component analysis (PCA) from household assets as an estimate of household socioeconomic status, and the tertiles of the scores used to define three groups (high, medium and low wealth ranking). The household items used to generate the wealth quintiles include ownership of large items such as land, car, to smaller items such as fan, radio, etc. Coverage estimates were obtained using standard survey methods. Survey-weighted coverages of 1st-year-of-life EPI antigens were calculated among children aged 12–23 months. Coverage of MCV Dose 2 was calculated in children aged 24–35 months (a 12-month age cohort beginning 6 months after the scheduled administration at 18 months of age). Vaccination status and receipt of vitamin A were determined from home-based records (HBR). Children were deemed vaccinated if the HBR was available and dates were recorded, regardless of the validity of these dates with respect to the child’s age. Children were considered unvaccinated if the HBR was available; however, the dates were not recorded. Denominators excluded children without an HBR. Children who received IPV at 14 weeks were deemed to have received OPV3. Children were considered fully vaccinated if they received all the recommended basic EPI vaccines by the time of the survey (BCG at birth, OPV × 3 (excluding birth dose), Pentavalent × 3, and one dose of measles. The malaria prevalence strata variable was generated by calculating the malaria prevalence in each cluster and grouped into three strata: low, medium, and high. Survey Poisson regression was used to estimate prevalence ratios.

## Results

### Background characteristics of participants

Overall, 7768 children aged 5–48 months from 6778 households participated in the survey. Table 1 describes the background characteristics of the children included in the analysis, comparing them with those in implementing and comparator districts. There were slightly more males (51.6%) than females, and nearly 52% of the children were residents of rural areas, with 29% being aged 12–23 months. There was almost an equal number of children in the comparator (3887) and intervention (3881) districts, as well as in each wealth index category.

**Table 1:** Background characteristics of children at baseline in MVIP regions of Ghana, 2019

Overall	Comparator n (%)	Intervention n (%)	Total n (%)
Age group			
5–11 months	729 (18.8)	729 (18.8)	1458 (18.8)
12–23 months	1176 (30.3)	1079 (28)	2255 (29.2)
24–35 months	1037 (27.3)	1098 (28.6)	2135 (27.9)
36–48 months	945 (23.6)	975 (24.6)	1920 (24.1)
Gender of child			
Male	1968 (50.7)	2012 (52.5)	3980 (51.6)
Female	1919 (49.3)	1869 (47.5)	3788 (48.4)
Residence			
Urban	1731 (48.8)	1657 (48.3)	3388 (48.5)
Rural	2156 (51.2)	2224 (51.7)	4380 (51.5)
Malaria prevalence^a^			
Low	973 (27.6)	1665 (35.9)	2638 (31.8)
Medium	1733 (42.5)	821 (30.3)	2554 (36.4)
High	1181 (29.9)	1395 (33.8)	2576 (31.8)
Wealth Index			
Low	1175 (27)	1420 (37.6)	2595 (32.4)
Medium	1337 (33.7)	1251 (31.8)	2588 (32.7)
High	1375 (39.3)	1210 (30.6)	2585 (34.9)
RDT Malaria test			
Negative	3056 (79.6)	3076 (79.3)	6132 (79.4)
Positive	831 (20.4)	805 (20.7)	1636 (20.6)
ITN use			
Didi not sleep under ITN last night	1434 (40.2)	1360 (36.6)	2794 (38.4)
Slept under net last night	2453 (59.8)	2521 (63.4)	4974 (61.6)
Total	3887 (100)	3881 (100)	7768 (100)

^a^Malaria prevalence was generated by calculating the malaria prevalence in each cluster and grouped into 3 strata rather than using defined prevalence cutoffs

### Malaria prevalence

Table 2 describes the prevalence of malaria by background characteristics. The survey was conducted during the low transmission season for malaria in Ghana. Using RDT, the prevalence of malaria among all children aged 5–48 months was 21% [95% CI (18–23)]; 20% [95% CI (17–24)] in the comparator, and 21% [95% CI (18–24)] in the implementing districts. Malaria prevalence was higher in rural than urban areas, with a prevalence ratio (PR) of 2.04 (95% CI 1.56–2.68). Children from households with a high wealth index had lower malaria prevalence than those from households with a low wealth index; PR = 0.37; 95% CI (0.30–0.44). Among children aged 36–48 months, 24% [95%CI (21–27)] tested positive for malaria parasitemia compared to 13% [95%CI (11–15)] among those aged 5–15 months. Malaria prevalence was lower among females than among males (PR = 0.91; 95% CI 0.82–1.00). There was no difference in malaria prevalence by ITN use.

**Table 2:** Malaria prevalence by background characteristics of children aged 5–48 in the MVIP regions of Ghana, 2019

Variable	Number of children (unweighted)	Number of positive cases (unweighted)	Malaria prevalence % (95% CI) (weighted)	Prev Ratio (95% CI) (weighted)
Overall	7768	1636	21 (18, 23)	
Age group				
5–11 months	1458	186	13 (11, 15)	Reference
12–23 months	2255	433	19 (17, 22)	1.53 (1.29, 1.80)
24–35 months	2135	527	25 (22, 28)	1.94 (1.64, 2.30)
36–48 months	1920	490	24 (21, 27)	1.88 (1.58, 2.23)
Gender of child				
Male	3980	869	22 (19, 24)	Reference
Female	3788	767	20 (17, 22)	0.91 (0.82, 1.00)
Residence				
Urban	3388	447	13 (11, 17)	Reference
Rural	4380	1189	27 (24, 31)	2.04 (1.56, 2.68)
Wealth Index				
Low	2595	761	29 (26, 32)	Reference
Medium	2588	586	23 (20, 26)	0.78 (0.68, 0.89)
High	2585	289	11 (09, 13)	0.37 (0.30, 0.44)
ITN use				
Did not sleep under ITN last night	2794	564	21 (18, 24)	Reference
Slept under ITN last night	4974	1072	20 (18, 23)	0.99 (0.85, 1.14)
Randomization Arm				
Comparator area	3887	831	20 (17, 24)	Reference
Implementation area	3881	805	21 (18, 24)	1.01 (0.82, 1.26)

### Malaria interventions: ownership and usage of mosquito nets

Overall, 87%, 95%CI (85–89) of households in the survey (87%; 95%CI (85–89) in implementation and 86%; 95%CI (84–88) in comparator districts) owned at least one mosquito net (Table 3). More households in rural [90% 95%CI (88–91)] than urban areas [83% 95%CI (81–85)] owned at least one mosquito net; PR = 1.08, 95%CI (1.04–1.11). Ownership was 84%, 95%CI (82–86%) among households in the poorest quintile, while 88%, 95%CI (86–90%) of households in the wealthiest quintile had at least one mosquito net.

**Table 3:** Household usage and ownership of insecticide-treated nets by background characteristics in MVIP regions of Ghana, 2019

	Usage of ITN		
Background characteristic	Number of children who slept under ITN a night before the survey	% (95%CI) weighted	Prev Ratio (95%CI) weighted
Overall	4974	62 (59, 64)	
Age group			
5–11 months	974	66 (62, 69)	Reference
12–23 months	1506	64 (61, 67)	0.97 (0.91, 1.03)
24–35 months	1325	60 (56, 63)	0.90 (0.85, 0.96)
36–48 months	1169	57 (54, 61)	0.87 (0.81, 0.93)
Gender of child			
Male	2548	62 (59, 65)	Reference
Female	2426	61 (58, 63)	0.97 (0.93, 1.02)
Residence			
Urban	1861	53 (49, 58)	Reference
Rural	3113	69 (66, 72)	1.30 (1.18, 1.43)
Randomization arm			
Comparison	2453	60 (57, 63)	Reference
Implementation	2521	63 (60, 67)	1.06 (0.98, 1.15)
Malaria prevalence			
Low	1535	57 (53, 62)	Reference
Medium	1706	63 (58, 68)	1.10 (0.99, 1.23)
High	1733	64 (61, 68)	1.13 (1.02, 1.24)
Wealth index			
Low	1723	64 (60, 67)	Reference
Medium	1695	63 (60, 67)	1.00 (0.93, 1.06)
High	1556	58 (55, 62)	0.92 (0.85, 0.98)

Ownership of ITN^a^			
	No. of households with at least one ITN	% (95%CI) weighted	Prev Ratio (95%CI) (weighted
Overall	5904	87 (85, 88)	
Residence			
Urban	2450	83 (81, 85)	Reference
Rural	3454	90 (88, 91)	1.08 (1.04, 1.11)
Randomization arm			
Comparison	2998	87 (85, 89)	Reference
Implementation	2906	86 (84, 88)	0.99 (0.85, 1.02)
Wealth index			
Low	1915	84 (82, 86)	Reference
Medium	1967	87 (84, 88)	1.03 (1.00, 1.06)
High	2022	88 (86, 90)	1.05 (0.82, 0.87)

^a^Unit of analysis for ITN ownership was the household; for ITN use, individual children aged 5 to 48 months

On the night before the survey, 62% (95% CI 59–64) of children aged 5–48 months slept under an ITN, compared to 60% (95% CI 57–63) in the same period, and 63% (95% CI 60–67) in implementation districts (Table 3). This percentage was higher in rural than urban areas; PR = 1.30, 95%CI (1.18–1.43). Children in the age group of 24–35 months and 36–48 months were less likely to sleep under an ITN compared to children aged 5–11 months. The proportion of children who slept under an ITN the night before the survey from high socioeconomic status households was lower than that among those from low socioeconomic status households, PR = 0.92, 95%CI (0.85–0.98). About 64% (61–68) of children from high malaria prevalence areas, compared to 57% (53–62) from low malaria prevalence areas, slept under an ITN the night before the survey, PR = 1.13, 95%CI (1.02–1.24).

### Health-seeking behaviour for febrile illness

In all, 1723 out of the 7,768 children aged 5–48 months [22%, 95%CI (21–24)] reported having fever in the two weeks prior to the survey. (Table 4) Among those with reported fever, 72%, 95%CI (69–74) sought advice or treatment, 40%, 95%CI (37–44) were tested for malaria, and 42%, 95%CI (39–46) took an antimalarial drug. Among children with reported fever in the two weeks preceding the survey, 12% (95% CI 10–14) reported taking an artemisinin-based combination therapy (ACT), and 5% (95% CI 4–6) took an ACT within 48 h of the onset of symptoms. Health-seeking behaviour for febrile illness was similar between implementation and comparator areas (Supplementary Fig. 1).

**Table 4:** Health-seeking behaviour by background characteristics in the MVIP regions of Ghana, 2019

	Children with fever in past 2 weeks	Children who sought advice or treatment	Children who reported having finger or heel stick	Children who took an antimalarial drug^a^	Children with fever who took ACT	Children with fever that took ACT within 48 h
Background characteristic	% (95%CI)	% (95%CI)	% (95%CI)	% (95%CI)	% (95%CI)	% (95%CI)
Overall	[1723/7768] 22(21,24)	[1192/1723] 72(69,74)	[693/1723] 40(37,44)	[603/1462] 42(39,46)	[201/1723] 12(10,14)	[67/1723] 5(4,6)
Age group						
5–11 months	[278/1458] 18(16,21)	[183/278] 66(59,72)	[98/278] 31(24,39)	[73/241] 33(27,41)	[21/278] 7(4,11)	[8/278] 3(2,6)
12–23 months	[540/2255] 26(23,28)	[387/540] 75(71,79)	[220/540] 42(37,47)	[176/461] 41(34,48)	[51/540] 10(7,13)	[15/540] 3(2,6)
24–35 months	[507/2135] 24(22,28)	[348/507] 71(67,76)	[213/507] 42(37,48)	[196/429] 45(40,50)	[69/507] 14(11,18)	[25/507] 6(4,9)
36–48 months	[398/1920] 20(18,22)	[274/398] 70(64,75)	[162/398] 41(34,48)	[158/331] 48(42,55)	[60/398] 16(12,21)	[19/398] 7(4,11)
Gender of child						
Male	[891/3980] 23(21,25)	[617/891] 71(68,75)	[357/891] 40(36,44)	[315/755] 44 (40, 48)	[107/891] 12(10,15)	[32/891] 5(3,7)
Female	[832/3788] 22(20,24)	[575/832] 72(68,76)	[336/832] 40(36,45)	[288/707] 41(36,46)	[94/832] 11(9,14)	[35/832] 5(3,7)
Residence						
Urban	[684/3388] 21(19,24)	[515/684] 77(74,80)	[285/684] 42(37,48)	[211/603] 37(33,42)	[60/684] 9(7,12)	[27/684] 5(3,8)
Rural	[1039/4380] 23(21,26)	[677/1039] 6(63,71)	[408/1039] 38(34,42)	[392/859] 47(43,52)	[141/1039] 14(11,17)	[40/1039] 5(3,7)
Randomization Arm						
Comparison	[795/3887] 21(19,23)	[538/795] 72(68,75)	[317/795] 42(37,48)	[263/681]41(36,46)	[84/795] 10(8,13)	[33/795] 4(3,7)
Implementation	[928/3881] 24(22,26)	[654/928] 72(68,75)	[376/928] 38(35,43)	[340/781] 44(40,49)	[117/928] 13(11,16)	[34/928] 5(3,7)
Malaria prevalence						
Low	[551/2638] 20(18,23)	[406/551] 74(69,79)	[220/551] 38(33,44)	[162/479] 34(30,39)	[58/551] 11(8,14)	[26/551] 6(4,9)
Medium	[536/2554] 21(19,24)	[367/536] 75(72,79)	[231/536] 47(41,54)	[174/477] 40 (34,47)	[48/536] 11(8,14)	[22/536] 5(3,8)
High	[636/2576] 26(24,28)	[419/636] 66(62,71)	[242/636] 35(31,40)	[267/506] 53 (47,59)	[95/636] 14(10,18)	[19/636] 4(2,7)
Wealth index						
Low	[621/2595] 23(21,26)	[395/621] 66(61,71)	[223/621] 36(31, 41)	[206/508] 43 (38,48)	[73/621] 12(9,16)	[16/621] 3(2,7)
Medium	[570/2588] 22(20,24)	[411/570] 76(71,80)	[228/570] 41(36, 47)	[198/484] 40 (35,47)	[60/570] 11(8,14)	[23/570] 5(3,7)
High	[532/2585] 21(19,24)	[386/532] 73(69,78)	[242/532] 44(39, 49)	[199/470] 44 (39,51)	[68/532] 13(10,17)	[28/532] 6 (4,9)

^a^261 children with reported fever two weeks before the survey had missing values on whether they took an antimalarial drug

Although the proportion of children who reported having a fever two weeks prior to the survey was lower in urban areas than in rural areas, the proportion who sought treatment for the fever was higher in urban than in rural communities, PR = 1.14; 95% CI (1.06–1.23). Among children from high socioeconomic households, 44% (95% CI 39–49) underwent a malaria test, compared to those from low socioeconomic households (36%), 95% CI (31–41). Additionally, 47% (95% CI 43–52) of children in urban areas took an antimalarial drug for their fever, compared to 37% (95% CI 33–43) in rural communities, PR = 1.27, 95% CI (1.07–1.51).

### Vaccination coverage

Among the 2255 children aged 12–23 months, 1988 (88%) had vaccination cards. Among these 1988 children, coverage for BCG, the third dose of diphtheria and tetanus toxoids and pertussis–containing vaccine (DTP3), the third dose of polio vaccine, and the first dose of measles-containing vaccine (MCV1) was 93%, 95%, 95%, and 90%, respectively. Coverage for the third dose of PCV and the second dose of Rotavirus vaccine was 95% and 96% respectively. The second dose of measles vaccination coverage among children aged 24–35 months was 80%. Full vaccination coverage (BCG, measles, and three doses each of pentavalent (DPT-HepB-Hib) and polio vaccine (excluding polio vaccine given at birth) was 79%. The vaccination coverage of routine EPI vaccines, by participant background characteristics, is presented in Table 5. Full vaccination coverage was similar by sex, implementing and comparison districts, and wealth index. Full vaccination coverage (basic antigens) among children aged 12–23 months was higher in rural areas (81%) than in urban areas (76%), PR = 1.06, 95% CI (1.00–1.14).

**Table 5:** Vaccination coverage of routine EPI vaccines by background characteristic among children aged 12–23 months in the MVIP regions of Ghana

Vaccine			Gender of child		Residence		Randomization arm		Malaria prevalence			Wealth Index		
	Dose	Overall	Male	Female	Urban	Rural	Comparison	Implementing	Low	Medium	High	Low	Medium	High
			% (95% CI) weighted	% (95% CI) weighted	% (95% CI) weighted	% (95% CI) weighted	% (95% CI) weighted	% (95% CI) weighted	% (95% CI) weighted	% (95% CI) weighted	% (95% CI) weighted	% (95% CI) weighted	% (95% CI) weighted	% (95% CI) weighted
BCG	1	93 (92, 95)	94 (92, 96)	92(90, 95)	93 (91, 95)	94 (92, 95)	95 (93, 96)	92 (90, 94)	92 (90, 95)	96 (94, 98)	92 (90, 95)	93 (90, 95)	94 (92, 96)	94 (91, 96)
Penta	1	99 (99,100)	99 (98, 00)	100(99,100)	99 (98, 100)	100 (99,100)	100 (99, 00)	99 (98, 100)	99 (98, 100)	99 (99, 100)	100(99,100)	99 (98, 00)	100 (99, 00)	99 (98, 100)
	2	97 (96, 98)	97 (95, 99)	97 (96, 99)	96 (94, 98)	98 (97, 99)	99 (98, 99)	96 (94, 98)	96 (95, 98)	98 (97, 99)	97 (96, 99)	96 (94, 99)	98 (96, 99)	98 (96, 99)
	3	95 (93, 96)	94 (92, 97)	95 (93, 97)	93 (91, 96)	96 (94, 98)	96 (95, 97)	93 (91, 96)	93 (91, 96)	95 (93, 97)	96 (94, 98)	93 (89, 97)	96 (94, 98)	96 (94, 97)
Polio (OPV)	0													
	1	99 (98,99)	98 (97,100)	99 (98,100)	98 (96,100)	99 (99,100)	99 (99, 100)	98 (96, 100)	98 (97, 99)	98 (97, 100)	99 (99, 00)	97 (95,100)	98 (97, 100)	99 (98, 100)
	2	94 (93, 96)	94 (92, 96)	95 (93, 96)	93 (91, 96)	96 (94, 97)	96 (94, 97)	93 (90, 95)	93 (91, 96)	95 (93, 97)	95 (93, 97)	92 (89, 95)	96 (94, 97)	95 (93, 98)
	3	91 (90, 93)	91 (89, 94)	92 (90, 94)	90 (87, 93)	93 (91, 95)	92 (90, 95)	90 (88, 93)	90 (87, 93)	92 (89, 94)	93 (90, 96)	89 (85, 93)	93 (91, 95)	93 (90, 95)
PCV	1	99 (99, 00)	99 (98, 100)	100 (99, 100)	99 (98,100)	99 (99, 100)	100 (99, 100)	99 (98, 100)	99 (98, 100)	99 (98, 100)	100 (99,100)	99 (98,100)	99 (98, 100)	99 (98, 100)
	2	97 (96, 98)	97 (95, 99)	98 (97, 99)	96 (94, 98)	98 (97, 99)	99 (98, 99)	96 (94, 98)	96 (94, 98)	98 (97, 99)	98 (96, 99)	96 (94, 98)	98 (97, 100)	98 (96, 99)
	3	95 (94, 96)	95 (93, 97)	96 (94, 97)	94 (92, 96)	96 (95, 98)	96 (95, 97)	94 (92, 96)	93 (91, 96)	95 (94, 97)	96 (95, 98)	93 (90, 96)	96 (95, 98)	96 (94, 97)
Rotavirus	1	99(98, 100)	99 (98, 100)	99 (99,100)	99 (98, 00)	99 (99, 100)	99 (99, 100)	98 (97, 100)	99 (98, 100)	99 (98, 100)	100(99,100)	98 (97, 00)	99 (98, 100)	99 (98, 100)
	2	96 (95, 97)	95 (93, 97)	97 (95, 98)	95 (93, 96)	97 (95, 98)	97 (96, 98)	94 (92, 96)	94 (92, 96)	96 (95, 98)	96 (95, 98)	94 (92, 96)	97 (96, 98)	96 (94, 98)
Measles	1	90 (89, 92)	90 (87, 92)	92 (89, 94)	87 (84, 90)	94 (92, 96)	91 (88, 93)	90 (88, 93)	88 (84, 91)	91 (89, 94)	92 (90, 95)	90 (87, 94)	91 (88, 94)	91 (88, 93)
	2	82 (79, 84)	82 (78, 85)	82 (78, 85)	76 (72, 81)	87 (84, 89)	80 (76, 85)	83 (80, 86)	80 (76, 84)	82 (78, 86)	82 (78, 86)	83 (79, 87)	82 (77, 87)	80 (74, 86)
^a^Fully vaccinated (Basic antigens)		79 (76, 81)	78 (75, 81)	80 (77, 83)	76 (72, 80)	81 (78, 85)	80 (77, 83)	77 (74, 81)	75 (71, 79)	81 (78, 85)	80 (76, 83)	76 (71, 80)	82 (78, 85)	79 (75, 83)

^a^To be fully vaccinated (basic antigens), a child must receive at least one dose of BCG, 3 doses of polio vaccine (OPV), IPV or a combination of OPV and IPV, 3 doses of Penta, and one dose of measles

### Vitamin A supplementation and deworming

Uptake of VAS in the past 6 months among children aged 6–48 months was 36% and coverage of deworming among children aged 12–48 months was 29% (Supplementary Table 1). About 39% of children in rural areas took VAS in the past six months compared to 29% in urban areas, PR = 1.35, 95%CI (1.19, 1.52). In addition, VAS uptake was lower among children aged 36–48 months (13%) compared to those aged 12–23 months (54%), PR = 0.24, 95% CI (0.19, 0.30). Deworming coverage among children aged 12–48 months was 24% in rural areas compared to 33% in urban settings, PR = 0.73, 95%CI (0.62, 0.86). Deworming coverage was higher among children from high socioeconomic households (36%) compared to those from low socioeconomic households (21%), PR = 1.69, 95% CI (1.46, 1.96). Similarly, deworming coverage was 35% among children aged 36–48 months and 20% among those aged 12–23 months, PR = 1.78, 95%CI (1.57, 2.02).

### Malnutrition

Overall, 17% (1257/7676) of children aged 5–48 months were at risk of malnutrition (MUAC ≤ 13.5 cm), 4% (295/7676) had moderate acute malnutrition (11.5 cm < MUAC ≤ 12.5 cm) and 1% (45/7676) had severe acute malnutrition (MUAC ≤ 11.5 cm). (Supplementary Table 2). Thirty-two percent of infants and 5% of children aged 36–48 months were at risk of malnutrition, PR = 0.16, 95%CI (0.13, 0.21). About 19% of girls, compared to 16% of boys, were at risk of malnutrition, PR = 1.22, 95%CI (1.11, 1.35). Additionally, 24% of children in clusters with a high prevalence of malaria, compared to 12% of children in clusters with a low prevalence of malaria, were at risk of malnutrition, PR = 2.00, 95%CI (1.68, 2.39) (Supplementary Table 2). The moderate acute malnutrition rate was 5% among girls and 3% among boys, PR = 1.52, 95%CI (1.21, 1.91). Similarly, 7% of children in clusters with high malaria prevalence had moderate acute malnutrition compared to 2% of children in clusters with low malaria prevalence, PR = 2.79, 95%CI (1.94, 4.03).

## Discussion

In this study, key relevant features of the area used for the malaria vaccine pilot evaluation in Ghana were described. Coverage of routine EPI vaccines, malaria prevalence, malaria control measures, VAS, and deworming coverage, as well as malnutrition status and patterns of health-seeking behavior for febrile children in the MVPE pilot regions of Ghana were estimated prior to the introduction of RTS,S. Overall, 22% of the children aged 5–48 months in our study tested positive for malaria. This is similar to the 17% malaria prevalence among children aged 6–59 months, as reported by RDT in the 2022 Ghana Demographic and Health Survey (GDHS) (13). Of note, children from poor households were more likely to test positive for malaria than those from wealthy households. This finding is consistent with those from Gambia and the 2022 GDHS, which showed that children in the fourth and richest quintiles were less likely to have malaria compared to those in the poorest quintiles [13, 14].

This study showed that 87% of households in the pilot regions owned at least one ITN, and 62% of children aged 5–48 months slept under an ITN the night before the baseline survey. One of the strategic objectives of the National Malaria Elimination Strategic Plan (NMESP) is to ensure that 100% of the population use at least one malaria preventive measure by 2028. Although a few years remain to achieve this, substantial improvement is still needed. The 2022 GDHS reported household ITN ownership at 67%, with 49% of children under the age of 5 sleeping under an ITN the night before the survey [13]. Although this study showed ownership and usage rates to be higher than those reported in the GDHS, a closer examination of the GDHS results revealed that ownership and usage rates were relatively high in the MVIP regions included in this study. Other studies in Ghana reported ownership of ITN of over 80% [15–17]. The higher ITN use in rural areas compared to urban areas seems likely to be appropriate, given the higher malaria transmission in rural areas, and likely reflects the subnational targeting of elimination efforts by the NMEP. The higher use in rural areas has also been noted in the GDHS and other studies. [13, 16, 18]. A survey in one of the pilot regions reported ITN usage of 66% [16]. A recent review of studies reported substantial gaps between ITN ownership and usage [19], highlighting the need to improve usage rates. In our survey, there was no difference in ITN ownership and usage between implementing and comparator clusters at baseline. This study showed that the children who slept under an ITN the night before the survey did not have a lower risk of malaria. This was unexpected, as many studies have shown a clear association between sleeping under an ITN and a reduction in the risk of malaria infection. However, considering that the baseline study was conducted in the low malaria transmission season in Ghana, low vector density might have masked the protective effect of ITN use. Additionally, this study did not examine the effect of other factors such as damaged or bio-efficacy of ITN, outdoor and early-evening mosquito bites which could have reduced the protective effect of ITN use.

In this study, 22% of all the participants reported having a fever in the 2 weeks preceding the survey. This finding is consistent with similar observations in Togo [20], but higher than the 15% reported in the GDHS [13] and lower than the rate observed in Malawi [21]. Advice or treatment was sought for 72% of all children with reported fever in the two weeks preceding the survey. Similar results were found in India [22] and Burkina Faso [23]. In the GDHS, 57% with fever sought advice or treatment [14]. In this study and the GDHS, 40% of febrile children had blood taken from a finger or heel for testing. This was higher than what was observed in Nigeria [24], and lower than what was found in Madagascar [25]. Additionally, one out of three febrile children were treated with ACT, and 5% of them received ACT within 48 h after the onset of symptoms. Our findings are consistent with a similar study in Senegal, where 6% of all febrile children received ACT within 48 h [26]. In another study in Sierra Leone, 48% of all febrile children received ACT, and 19% received ACT within 24 h [27]. Our findings, along with those of others, highlight the need to improve timely treatment with ACT for malaria.

Before the pilot implementation of the RTS,S/AS01 vaccine, there was no difference in the coverage of the routine EPI vaccines by the implementing and comparator clusters in Ghana. Coverage of BCG, the third dose of Penta, oral polio vaccine (OPV)/IPV, and the first dose of measles vaccine was all more than 90%. These coverages are comparable to the coverages reported in the 2021 GDHS [13]. Full vaccination coverage (basic antigens) was 79% in our study, similar to the 75% reported in the GDHS. Full vaccination coverage was higher in rural than urban areas. This contrasts with findings of the 2021 GDHS [13], and other African countries where full vaccination coverage was higher in urban than rural areas [28]. Our observation likely reflects the successful penetration of primary healthcare in rural Ghana through the implementation of the Community-based Health Planning and Services (CHPS), and rural outreach activities of Ghana’s EPI programme. Whilst coverage is based on children with HBR in this study, the coverage from GDHS is based on both HBR and maternal recall.

Deworming and VAS coverages were similar in implementing and comparator clusters The proportion of children aged 6–48 months who received VAS in the last 6 months (36%), and those aged 12–48 months given deworming during the previous 6 months (29%) were lower than what was observed in the 2022 GDHS report [13]. In East Africa, the prevalence of deworming 6 months before the interview was 54% [29]. Consistent with our observations, deworming coverage was higher in rural areas than in urban areas, and also higher among children from the wealthiest households than from the poorest families [29]. However, VAS coverage was higher in rural than urban areas. This is consistent with observations elsewhere in sub-Saharan Africa [30].

Although progress is being made to reduce malnutrition in Ghana, it remains a significant health issue. In this study, 17% of children were at risk of malnutrition in both the implementing and comparator clusters. Nearly 1% of participants had severe acute malnutrition (SAM), which is consistent with a similar study that reported a 1.4% SAM rate in northern Ghana [31]. Other studies reported a severe malnutrition prevalence of 2.4% [32]. The 2022 GDHS indicated that 17% of children under age five were stunted and 6% were wasted [13]. Another study in Ghana reported that almost 36% of children under 5 years of age suffered from some form of malnutrition, and 9% were wasted [33].

It is essential to note that some of the responses in this study, such as receipt of deworming medication six months prior to the interview and having a fever two weeks prior to the survey day, were based on self-report and may have been affected by recall bias. Secondly, the reporting of fever by caregivers was not validated by medical examination. This study was conducted between February and March 2019, which is the low malaria transmission season in Ghana. Therefore, some indicators, such as reported fever prevalence and health-seeking behaviours, may differ substantially during the high malaria transmission season. The findings of this study should be interpreted in light of the season during which the study was conducted. Despite these limitations, the study has considerable strengths. First, we used data from a large population-based survey. Second, the survey used some of the questions from the Demographic and Health Surveys to ensure comparability of results. Third, the survey was rigorously supervised and covered key areas, including malaria control measures, malaria prevalence, immunizations, deworming, VAS coverage, malnutrition, and health-seeking behaviors for febrile children. Fourth, this study generated robust baseline data to facilitate the evaluation of the feasibility, safety, and impact of RTS,S when implemented through the national immunization programme [9].

## Conclusions

Despite recent reductions in malaria prevalence nationally, 21% of children aged 5–48 months in this survey tested positive for malaria parasitaemia by RDT. While 87% of households owned at least one ITN, only 62% of children slept under an ITN the night before the survey. Of the 22% of participants with reported fever in the two weeks preceding the survey, 40% were tested for malaria. Coverage of the routine EPI vaccines was high, and 17% of children were at risk of malnutrition. Malaria prevalence, use of ITNs, health-seeking behaviour for febrile children, malnutrition rates, vaccination coverages, VAS, and deworming uptake were similar in implementing and comparator districts at baseline. This study has effectively established the baseline comparability between implementation and comparator areas, which serves as a foundation for future feasibility assessments.

## Supplementary Information

Supplementary Material 1

## Data Availability

Anonymized data will be made available through Harvard Dataverse. A Data Access Committee will review access requests.

## Abbreviations

ACT: Artemisinin-based combination therapy
EPI: Expanded Programme on Immunisation
GDHS: Ghana Demographic and Health Survey
GHS: Ghana Health Service
GSS: Ghana Statistical Service
IPTp: Intermittent Preventive Treatment in pregnancy
IRS: Indoor Residual Spraying
ITN: Insecticide–Treated Net
LLINs: Long-Lasting Insecticidal Nets
MVPE: Malaria Vaccine Pilot Evaluation
RDT: Rapid diagnostic test
SP: Sulfadoxine–pyrimethamine
SSA: Sub-Saharan Africa
SMC: Seasonal Malaria Chemoprevention
VAS: Vitamin A supplementation
