# Supplementary material for: The Malaria Vaccine Implementation Programme study area in Ghana: results of a household survey prior to the introduction of the RTS,S/AS01 vaccine
Source: Malar J. 2026 Jan 12;25:69. doi: 10.1186/s12936-025-05778-9 (PMC12862907; doi:10.1186/s12936-025-05778-9)
Supplement: Supplementary file 1 — Supplementary Material 1 [file 12936_2025_5778_MOESM1_ESM.docx]

**Supplementary Table 1: Coverage of Vitamin A Supplementation and Deworming by background characteristics in the MVIP regions of Ghana**

|  | **Vitamin A supplementation (VAS) coverage in the past 6 months among children aged 6-48 months (N=6,403)** | | | **Deworming coverage among children aged 12-48 months** | | |
| --- | --- | --- | --- | --- | --- | --- |
| **Background Characteristics** | **Number that received VAS** | **% (95% CI) weighted** | **Prev Ratio (95% CI) weighted** | **n (unweighted)** | **% (95% CI) weighted** | **Prev Ratio (95% CI) weighted** |
| **Overall** | 2280 | 35 (33, 36) |  | 1802 | 0.29 (0.27, 0.31) |  |
| **Age group** |  |  |  |  |  |  |
| 6-11 months | 580 | 54 (50, 58) | Reference |  |  |  |
| 12-23 months | 1023 | 50 (48, 53) | 0.92 (0.84, 1.01) | 451 | 20 (18, 22) | Reference |
| 24-35 months | 455 | 24 (22, 27) | 0.45 (0.39, 0.52) | 686 | 32 (30, 35) | 1.64 (1.45, 1.87) |
| 36-48 months | 222 | 13 (10, 16) | 0.24 (0.19, 0.30) | 665 | 35 (32, 38) | 1.78 (1.57, 2.02) |
| **Gender of child** |  |  |  |  |  |  |
| Male | 1159 | 35 (33, 37) | Reference | 922 | 28 (26, 31) | Reference |
| Female | 1121 | 34 (32, 37) | 0.98 (0.91, 1.06) | 880 | 29 (27, 31) | 1.01 (0.93, 1.10) |
| **Residence** |  |  |  |  |  |  |
| Urban | 800 | 29 (27, 32) | Reference | 940 | 33 (30, 37) | Reference |
| Rural | 1480 | 39 (37, 42) | 1.35 (1.19, 1.52) | 862 | 24 (18, 23) | 0.73 (0.62, 0.86) |
| **Randomization arm** |  |  |  |  |  |  |
| Comparison | 1122 | 34 (32, 36) | Reference | 853 | 28 (25, 31) | Reference |
| Implementation | 1158 | 35 (32, 38) | 1.04 (0.93, 1.16) | 949 | 29 (27, 32) | 1.03 (0.91, 1.18) |
| **Malaria prevalence** |  | | | | | |
| Low | 796 | 35 (31, 40) | Reference | 707 | 33 (30, 36) | Reference |
| Medium | 803 | 35 (32, 38) | 0.99 (0.86, 1.14) | 585 | 30 (27, 34) | 0.93 (0.80, 1.08) |
| High | 681 | 33 (30, 36) | 0.94 (0.81, 1.09) | 510 | 23 (20, 26) | 0.69 (0.58, 0.81) |
| **Wealth Index** |  |  |  |  |  |  |
| Low | 801 | 36 (33, 39) | Reference | 465 | 21 (19, 24) | Reference |
| Medium | 738 | 34 (32, 37) | 0.95 (0.85, 1.07) | 582 | 28 (25, 31) | 1.31 (1.13, 1.52) |
| High | 741 | 34 (31, 36) | 0.93 (0.83, 1.05) | 755 | 36 (33, 39) | 1.69 (1.46, 1.96) |

**Supplementary Table 2: Malnutrition by background characteristics in the MVIP regions of Ghana**

| **Variable** |  |  | **Moderate acute malnutrition (11.5cm<MUAC ≤ 12.5cm)** | | | **Severe acute malnutrition**  **(MUAC ≤ 11.5cm)** | | | **Risk of malnutrition**  **(MUAC ≤ 13.5cm)** | | |
| --- | --- | --- | --- | --- | --- | --- | --- | --- | --- | --- | --- |
|  | **n** | **Mean (SE)** | **n** | **% (95% CI)** | **Prev ratio** | **n** | **% (95% CI)** | **Prev ratio** | **n** | **% (95% CI)** | **Prev ratio** |
| Overall | 7676 | 14.92 (0.03) | 295 | 4 (4, 5) |  | 45 | 1 (0, 1) |  | 1257 | 17 (16, 19) |  |
| **Age group** |  |  |  |  |  |  |  |  |  |  |  |
| 5-11 | 1401 | 14.35 (0.05) | 106 | 8 (6, 10) | Reference | 18 | 1 (1, 2) | Reference | 425 | 32 (29, 35) | Reference |
| 12-23 | 2255 | 14.53 (0.05) | 127 | 7 (5, 8) | 0.84 (0.63, 1.11) | 21 | 1 (1, 2) | 0.82 (0.38, 1.77) | 527 | 25 (23, 28) | 0.80 (0.71, 0.90) |
| 24-35 | 2135 | 15.18 (0.05) | 40 | 2 (1, 3) | 0.25 (0.18, 0.39) | 5 | 0 (0, 0) | 0.14 (0.05, 0.39) | 214 | 10 (8, 12) | 0.31 (0.26, 0.37) |
| 36-48 | 1885 | 15.55 (0.05) | 22 | 1 (1, 2) | 0.14 (0.08, 0.25) | 1 | 0 (0, 0) | 0.01 (0.00, 0.08) | 91 | 5 (4, 7) | 0.16 (0.13, 0.21) |
| **Gender** |  |  |  |  |  |  |  |  |  |  |  |
| Boys | 3925 | 15.00 (0.04) | 121 | 3 (3, 4) | Reference | 21 | 1 (0, 1) | Reference | 577 | 16 (14, 17) | Reference |
| Girls | 3751 | 14.85 (0.04) | 174 | 5 (4, 6) | 1.52 (1.21, 1.91) | 24 | 1 (0, 1) | 1.05 (0.50, 2.20) | 680 | 19 (17, 21) | 1.22 (1.11, 1.35) |
| **Residence** |  |  |  |  |  |  |  |  |  |  |  |
| urban | 3359 | 14.97 (0.05) | 123 | 4 (3, 5) | Reference | 23 | 1 (0, 1) | Reference | 507 | 16 (14, 18) | Reference |
| rural | 4317 | 14.88 (0.04) | 172 | 4 (4, 5) | 1.11 (0.80, 1.56) | 22 | 1 (0, 1) | 0.85 (0.40, 1.80) | 750 | 18 (17, 20) | 1.16 (0.96, 1.40) |
| **Randomisation arm** |  |  |  |  |  |  |  |  |  |  |  |
| comparison | 3839 | 15.00 (0.05) | 168 | 5 (4, 6) | Reference | 24 | 1 (0, 1) | Reference | 646 | 17 (16, 19) |  |
| implementation | 3837 | 14.85 (0.03) | 127 | 4 (3, 5) | 0.82 (0.60, 1.11) | 21 | 1 (0, 1) | 0.93 (0.44, 1.97) | 611 | 17 (15, 19) | 0.97 (0.84, 1.12) |
| **Wealth index** |  |  |  |  |  |  |  |  |  |  |  |
| Lowest third | 2563 | 14.77 (0.06) | 123 | 5 (4, 7) | Reference | 24 | 1 (1, 2) | Reference | 489 | 20 (18, 22) | Reference |
| Middle | 2552 | 14.93 (0.04) | 88 | 4 (3, 5) | 0.79 (0.57, 1.09) | 13 | 0 (0, 1) | 0.47 (0.22, 1.01) | 407 | 18 (16, 20) | 0.89 (0.78, 1.02) |
| Highest third | 2561 | 15.07 (0.05) | 84 | 4 (3, 5) | 0.69 (0.48, 1.01) | 8 | 0 (0, 1) | 0.28 (0.10, 0.74) | 361 | 14 (13, 16) | 0.72 (0.60, 0.85) |
| **Malaria Prevalence** |  |  |  |  |  |  |  |  |  |  |  |
| Lowest third | 2607 | 15.13 (0.05) | 61 | 2 (2, 3) | Reference | 12 | 1 (0, 1) | Reference | 321 | 12 (11, 14) | Reference |
| Middle | 2521 | 15.02 (0.05) | 85 | 4 (3, 5) | 1.57 (1.13, 2.19) | 14 | 1 (0, 1) | 0.92 (0.32, 2.65) | 369 | 16 (14, 17) | 1.30 (1.10, 1.54) |
| Highest third | 2548 | 14.62 (0.05) | 149 | 7 (5, 9) | 2.79 (1.94, 4.03) | 19 | 1 (0, 1) | 1.17 (0.47, 2.91) | 567 | 24 (22, 27) | 2.00 (1.68, 2.39) |

Supplementary Figure 1: Health-seeking behaviour for febrile illness by implementation and comparator areas
